# Effects of Root Zone Warming on Maize Seedling Growth and Photosynthetic Characteristics Under Different Phosphorus Levels

**DOI:** 10.3389/fpls.2021.746152

**Published:** 2021-12-09

**Authors:** Zhenqing Xia, Shibo Zhang, Qi Wang, Guixin Zhang, Yafang Fu, Haidong Lu

**Affiliations:** College of Agronomy, Northwest A&F University, Shaanxi, China

**Keywords:** maize, root zone warmed, phosphate deficiency, stress physiology, photosynthesis

## Abstract

Phosphorus content and root zone temperature are two major environmental factors affecting maize growth. Both low phosphorus and root zone high temperature stress significantly affect the growth of maize, but the comprehensive effects of phosphorus deficiency and root zone warming are less studied. This study aimed to explore the effects of phosphorus deficiency and root zone warming on the root absorption capacity, total phosphorus content, and photosynthetic fluorescence parameters of maize seedlings. The results showed that maize shoots and roots had different responses to root zone warming and phosphorus deficiency. Properly increasing the root zone temperature was beneficial to the growth of maize seedlings, but when the root zone temperature was too high, it significantly affected the root and shoot development of maize seedlings. The root zone warming had a more significant impact on the root system, while phosphorus deficiency had a greater impact on the shoots. Phosphorus content and root zone warming had a strong interaction. Under the comprehensive influence of normal phosphorus supply and medium temperature in the root zone, the growth of maize seedlings was the best. Under the combined effects of low phosphorus and high temperature in the root zone, the growth was the worst. Compared with the combination of normal phosphorus and root zone medium temperature treatment, the dry mass of the low-phosphorus root zone high temperature treatment was decreased by 55.80%. Under the condition of low-phosphorus too high root zone temperature reduced root vitality, plant phosphorus content, which in turn affected plant growth and light energy utilization efficiency. In the case of sufficient phosphate fertilizer supply, appropriately increasing the soil temperature in the root zone is beneficial to increase the absorption and utilization of phosphorus by plants and promote the growth and development of maize seedlings.

## Introduction

Temperature is one of the key environmental factors affecting crop growth, and most crops are extremely sensitive to the ambient temperature. In recent years, the average temperature around the world has accelerated (Ekwurzel et al., 2017). IPCC (2013) evaluation report pointed out that the global average surface temperature in 2016–2035 may rise 0.3–0.7°C, expected to rise at the end of the 21st century, the surface temperature of the earth will increase by 1.4–5.8°C (Zhang et al., 2019). Soil temperatures are closely related to atmospheric temperatures, and the soil temperature near the root zone also increases in the background of the rise in temperature in the global temperature (Qian et al., 2011). The researchers conducted an investigation on the annual average temperature and soil temperature of 217 locations northern Eurasia during 1981–2015, and it was found that for the region as a whole, warming rate of soil temperature at 0.2m by 0.36±0.03°Cdecade^−1^ was slightly greater than the increase in air temperature (Chen et al., 2021). The continuous increase of the soil temperature in the root zone will certainly have an important impact on the crop growth.

Soil temperature is an important environmental factor for crop growth, and it is closely related to the development of root and shoot (Xia et al., 2021b). Suitable soil temperatures promote crop growth, while low or high temperatures increase soil adversity stress (Xia et al., 2021b; Ylivainio and Peltovuori, 2012). Soil temperature significantly affects the effectiveness of soil nutrients and moisture (Xia et al., 2021b; Ylivainio and Peltovuori, 2012). The decomposition rate of organic matter is different under different soil temperatures, and the activities of various enzymes and microorganisms related to crop growth in the soil are regulated by soil temperature (Behtari et al., 2019; Robinson et al., 2020). At the same time, the movement of soil solution and the form of soil water are also affected by soil temperature. When the soil temperature becomes higher, the movement of soil water becomes more frequent, the gaseous water in the soil increases, and the solid water available for crop roots decreases (Liu et al., 2020). When soil moisture is insufficient, higher soil temperature is likely to aggravate drought stress (Yin et al., 2016). Studies have found that the root absorption area and root vigor of crops are significantly reduced when the root zone temperature is too high, and the excessive accumulation of active oxygen in the roots leads to membrane lipid peroxidation and enzyme inactivation, which affects the function of root absorption and synthesis (Xia et al., 2021a; Xia et al., 2021b). The growth and development of crops above the ground are significantly related to the root system. The high temperature of the soil directly affects the root system, and at the same time indirectly affects the material and energy supply of the above ground, which in turn affects photosynthesis and dry mass accumulation (Xia et al., 2021a; Xia et al., 2021b). Phosphorus is an essential nutrient element for plant growth and is closely related to plant growth and development, material energy metabolism, and photosynthetic respiration (Zhang et al., 2016, 2018). During the growth of field crops, lack of phosphorus often leads to poor growth and development, which affects its yield and quality (Zhang et al., 2018; Wang et al., 2020). Although most soils are rich in phosphorus, most of them are recalcitrant phosphorus that cannot be absorbed and utilized by plants. Studies have shown that the phosphorus that can be absorbed by crops only accounts for 10–25% of phosphorus in the soil (Richardson and Simpson, 2011). Maize is very sensitive to phosphorus at the seedling stage. Phosphorus deficiency at the seedling stage will significantly reduce the phosphorus content of the plant and hinder the accumulation of dry mass. (Tang et al., 2019; Wang et al., 2020). Even if sufficient phosphorus is supplied in the later stage, it is difficult to remedy the loss caused by the earlier stage.

Soil temperature and phosphorus supply have a significant impact on the growth and development of maize. Soil temperature stress and phosphorus stress have similar effects on plants in some respects, and plants also have similar defense mechanisms. Oxidative stress caused by excessive accumulation of reactive oxygen species is one of the most important physiological factors affecting plant growth and development under adversity conditions (Foyer, 2018; Xia et al., 2021a). High temperature and phosphorus stress in the root zone can cause the accumulation of active oxygen in plants. In order to cope with the excessive accumulation of active oxygen, plants activate their oxidative defense mechanisms through enzymes or non-enzymes. For example, under environmental stress, superoxide dismutase and catalase can effectively detoxify or eliminate the accumulation of active oxygen, and osmotic regulators, such as proline, also play an important role (Liebthal et al., 2018). In addition, soil high temperature stress may destroy the plant’s low-phosphorus tolerance mechanism. For example, under low-phosphorus conditions, plants will increase the distribution of dry mass to the root system through the regulation of hormones, etc., to ensure the absorption of phosphorus by the root system, but too high soil temperature significantly reduces the development of the root system (Nacry et al., 2005; Xia et al., 2021b). A large number of studies have been carried out to investigate the effects of phosphorus content or soil temperature as a separate factor on plant growth, but the literatures on their combined effects are still limited (Zhang et al., 2018; Xia et al., 2021b). Plants exhibit a series of morphological and physiological adaptations under adversity stress, and different types of environmental stresses have a strong interaction (Woodrow et al., 2017; Tang et al., 2019). Tang et al. (2019) found that low phosphorus can improve salt tolerance under salt stress. Song et al. (2019) found that water and nitrogen had a significant coupling effect on maize seedling growth and root development, in addition, under drought stress conditions, nitrogen application would inhibit root development.

This experiment used hydroponics to simulate soil temperature changes by controlling the water temperature. In this experiment, we investigated the effects of root zone temperature increase and low-phosphorus stress on the root and shoot growth, plant phosphorus content, photosynthesis, and fluorescence parameters of maize seedlings. Trying to reveal the response of maize seedlings to soil warming and low-phosphorus stress and their interactions, and provide a theoretical basis for maize fertilization management measures under the background of climate warming.

## Materials and Methods

### Plant Growth

Seeds of maize (*Zea mays* L.) cv. SD609 (a local mainly cultivated cultivar), supplied from the Key Laboratory of Maize Biology and Genetics and Breeding in the Northwest Arid Region of the Ministry of Agriculture, were surface-sterilized in 70% (v/v) alcohol for 5min, soaked in distilled water for 12h, and then germinated in the dark on filter paper for 7days at 24°C. Subsequently, similar size seedlings were transferred to each plastic boxes (40cm×30cm×12cm) containing 10L of Hoagland nutrient solution. The nutrient solution consists of the following composition (μmol/L): Ca(NO_3_)_2_ (2000), MgSO_4_· 7H_2_O (650), K_2_SO_4_ (750), KCl (100), MnSO_4_·H_2_O (1), CuSO_4_·5H_2_O (0.1), ZnSO_4_ (1), (NH_4_)_6_Mo_7_O_24_ (0.05), Fe-ethylene diamine tetraacetic acid (10), and H_3_BO_3_ (1). Phosphorus was added to the nutrient as KH_2_PO_4_ at a concentration of 1.25μmol/L P (low P) and 250μmol/L P (normal P). Potassium was balanced by providing appropriate concentrations of KCl to the low-phosphorus treatments. The nutrient solutions were continually aerated and replaced every 3days. When the seedlings grew to the period of two leaves and one heart, put a variable frequency constant temperature heating rod (produced by Zhongbao Ribao Company, power 100W, temperature control accuracy ±0.2°C) in the plastic box for precise root zone temperature increase. The growth continued for 35days. Treatments were as follows:

1. 24°C water temperature (NT) with normal P (NP)
2. 24°C water temperature (NT) with low P (LP)
3. 30°C water temperature (MT) with normal P (NP)
4. 30°C water temperature (MT) with low P (LP)
5. 36°C water temperature (HT) with normal P (NP)
6. 36°C water temperature (HT) with low P (LP)

The experiment was a completely randomized design and there were three replicates for each treatment. The experiments were carried out in a 24°C culture room controlled by air conditioning with a light intensity of 300μmolm^−2^ s^−1^ and the electric air pump was used for continuous ventilation during the period. The plastic box was wrapped with sponge around and at the bottom, and the top was covered with a foam board with small holes to fix plants and reduce heat exchange.

### Determination of Dry Mass Accumulation and Root Morphology

After 35days of treatment, three plants were harvested for each plot, separated in shoot and root. Root was scanned with an EPSON V800 scanner (Epson China Co. Ltd., Beijing, China); data of root surface area, average root diameter, total root length, and root volume were analyzed by the Win RHIZO (Regent Instructions, Quebec, Canada) root system analysis software. Root and shoot dry mass were oven-dried (105°C for 15min and then 70°C for 72h) until constant weight and measured the dry mass with an electronic balance.

### Determination of Root Vitality and MDA Content

Malondialdehyde (MDA) content of root was determined by thiobarbituric acid method (Heath and Packer, 1968). Root samples (1g) were homogenized in 10ml 10% trichloroacetic acid. The homogenate was centrifuged at 4000rpm for 10min, and 2ml 0.6% thiobarbituric acid was added to a 2ml aliquot of the supernatant. The mixture was heated at boiling water bath for 15min and cooled rapidly. After centrifugation at 4000r/min for 10min, the absorbance was recorded at 532nm, 600nm, and 450nm. MDA concentration was calculated according to the equation:


$$C\left(\frac{\mu mol}{L}\right)=6.45\times \left(D532-D600\right)-0.56\times D450$$

where C is MDA concentration and D532, D600, and D450 represent the absorbance at 532nm, 600nm, and 450nm, respectively.

Root vitality was measured by triphenyltetrazolium chloride (TTC) method (Sturite et al., 2005). Root tip samples (0.5g) were submerged in 5ml 0.4% TTC and phosphate buffer at 37°C for 1h. Then, 2ml 1mol/L sulfuric acid was added to terminate reaction. And root samples were homogenized in ethyl acetate and quartz sand, the absorbance was recorded at 485nm.

### Determination of Phosphorus Content

The oven-dried root and shoot samples were ground to a fine powder and digested with a mixture of concentrated H_2_O_2_ and H_2_SO_4_. Root and shoot P concentration were measured by the vanado-molybdate method (Johnson and Ulrich, 1959).

### Determination of Photosynthetic Physiological Parameters

After 35days of treatment, the net photosynthetic rate (P_n_), transpiration rate (T_r_) of the last unfolded leaf of the maize seedlings was measured with a Li-6400 portable photosynthetic system analyzer (LI-COR in the United States).

### Determination of Chlorophyll Fluorescence

After 35days of treatment, the portable chlorophyll fluorometer (Dual-PAM 100, WALZ, Effeltrich, Germany) was used to determine the initial fluorescence (*F*_0_), maximal fluorescence (*F*_m_), minimum fluorescence (*F*_0_′), maximum fluorescence under light (*F*_m_′), and steady-state fluorescence (*F*_s_) of the last unfolded leaf of maize seedlings and the potential activity of PSII (*F*_υ_/*F*_0_)=(*F*_m_−*F*_0_)/*F*_0_, maximum quantum yield of PSII photochemistry (*F*_υ_/*F*_m_)=(*F*_m_−*F*_0_)/*F*_m_, actual photochemistry efficiency (Φ*_PSII_*)=(*F*_m_′−*F*_s_)/*F*_m_′, photochemical quenching coefficient (*qP*)=(*F*_m_′−*F*_s_)/(*F*_m_′−*F*_0_′), and non-photochemical quenching coefficient (NPQ)=1–*F*_m_′/*F*_m_.

### Statistical Analysis

**Figure 1:**
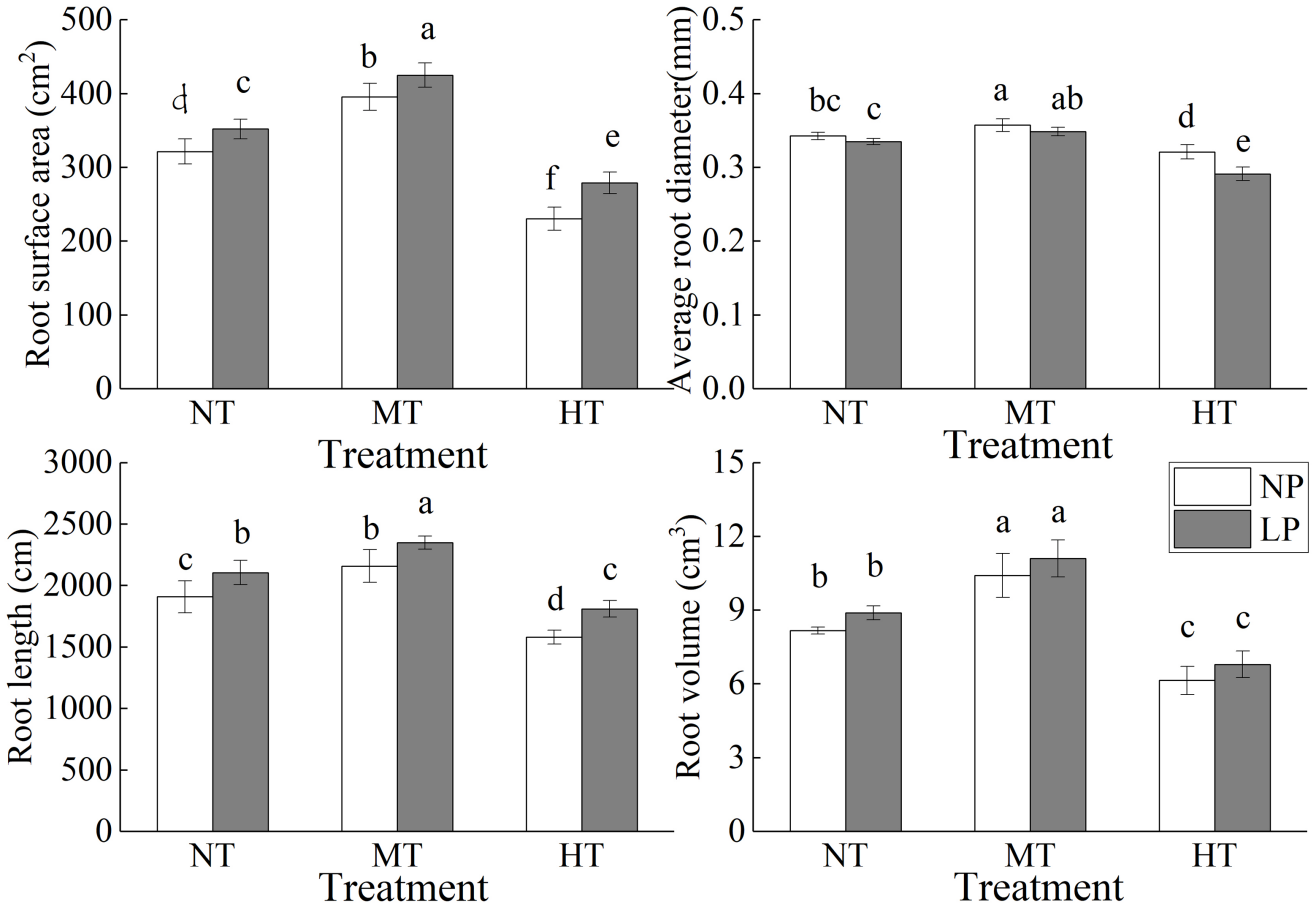
Effect of root zone temperature and phosphorus content on root surface area, average root diameter, root volume and total root length. NT: 24°C, MT: 30°C, and HT: 36°C (root zone temperature); LP: 1.25μm and NP: 250μm (phosphorus content). Notations above bars of a, b, c, d, e and f indicate homogeneous groups (*α*=0.05).

**Figure 2:**
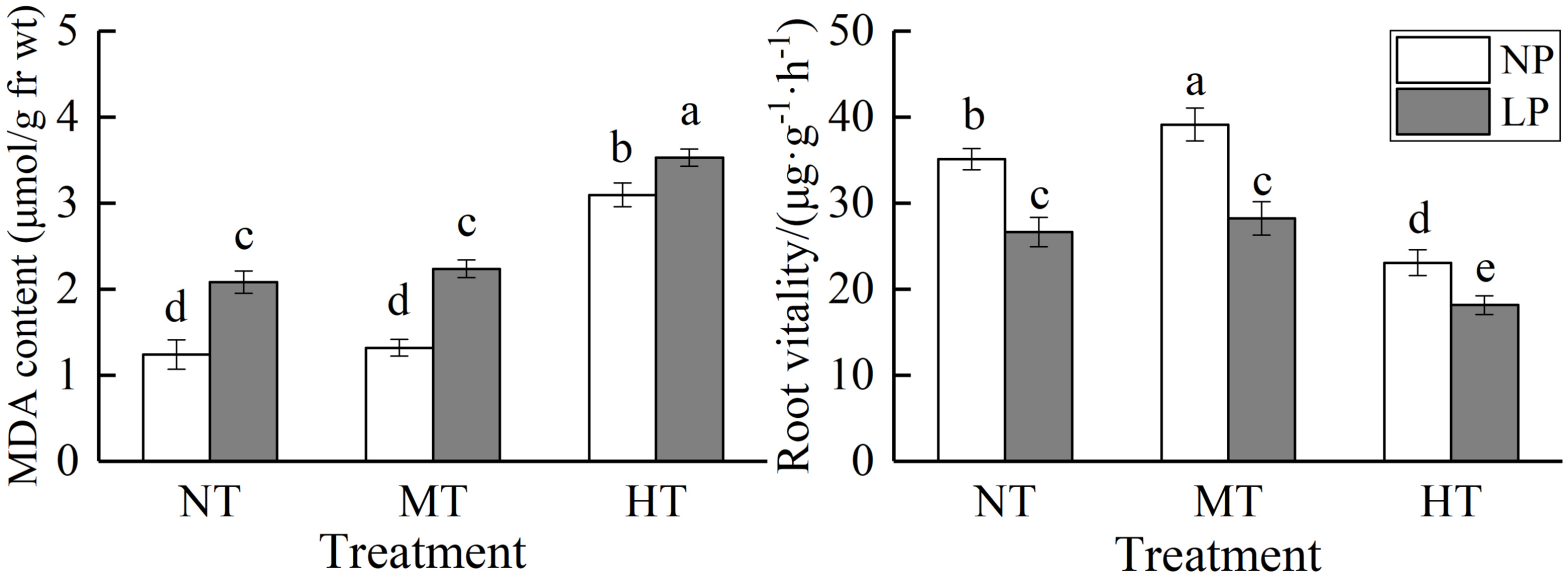
Effect of root zone temperature and phosphorus content on root MDA content and root vitality. NT: 24°C, MT: 30°C, and HT: 36°C (root zone temperature); LP: 1.25μm and NP: 250μm (phosphorus content). Notations above bars of a, b, c, d, and e indicate homogeneous groups (*α*=0.05).

**Figure 3:**
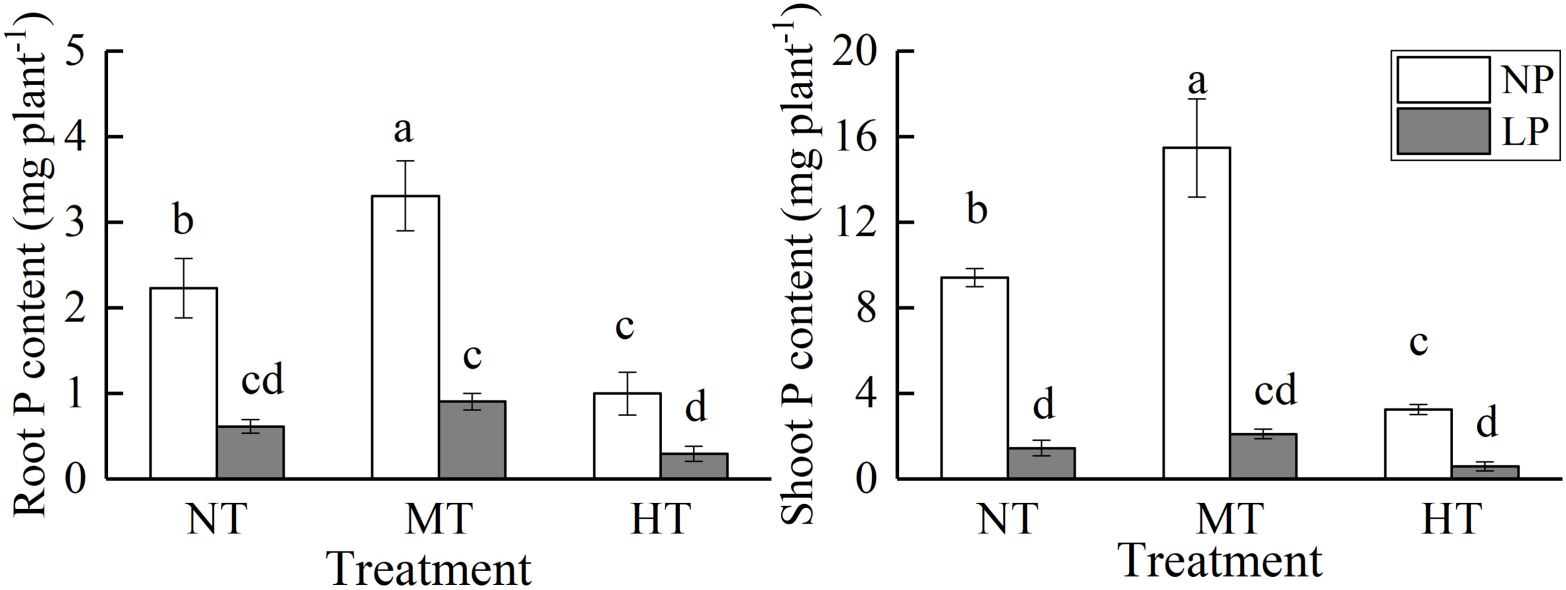
Effect of root zone temperature and phosphorus content on root and shoot phosphorus content. NT: 24°C, MT: 30°C, and HT: 36°C (root zone temperature); LP: 1.25μm and NP: 250μm (phosphorus content). Notations above bars of a, b, c, and d indicate homogeneous groups (*α*=0.05).

**Figure 4:**
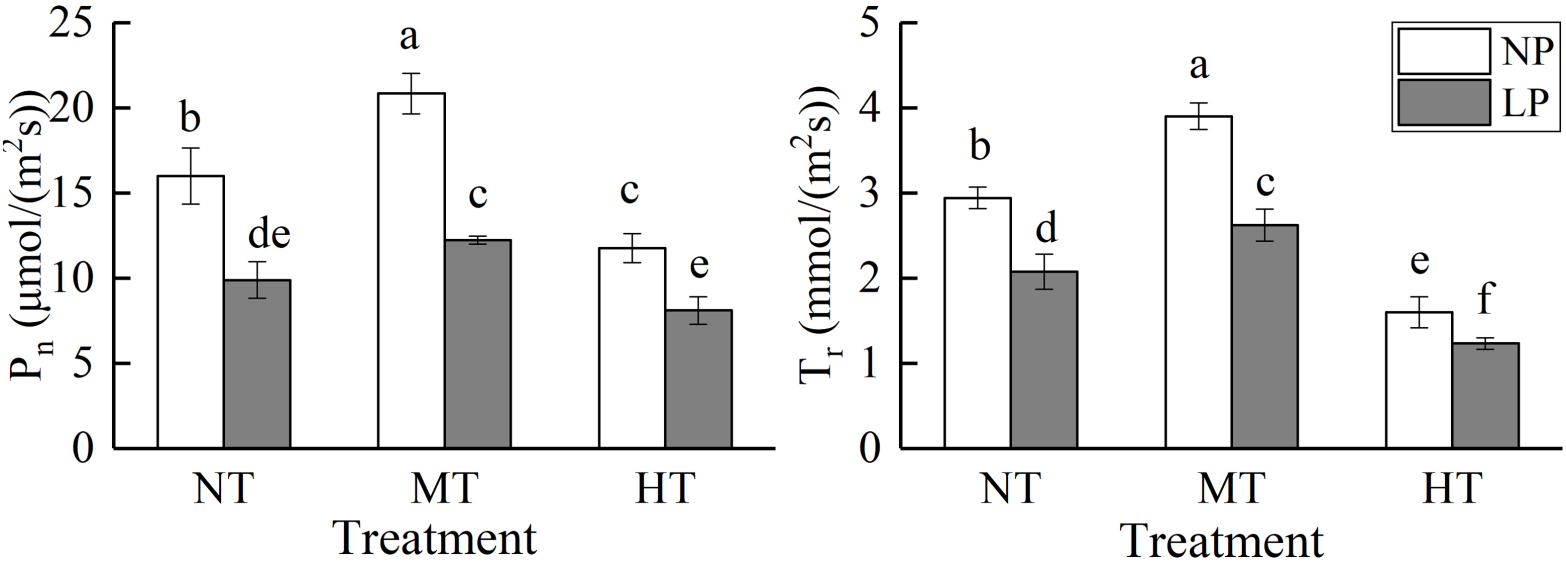
Effect of root zone temperature and phosphorus content on the net photosynthetic rate (Pn) and the transpiration rate (Tr). NT: 24°C, MT: 30°C, and HT: 36°C (root zone temperature); LP: 1.25μm and NP: 250μm (phosphorus content). Notations above bars of a, b, c, d, e, and f indicate homogeneous groups (*α*=0.05).

**Figure 5:**
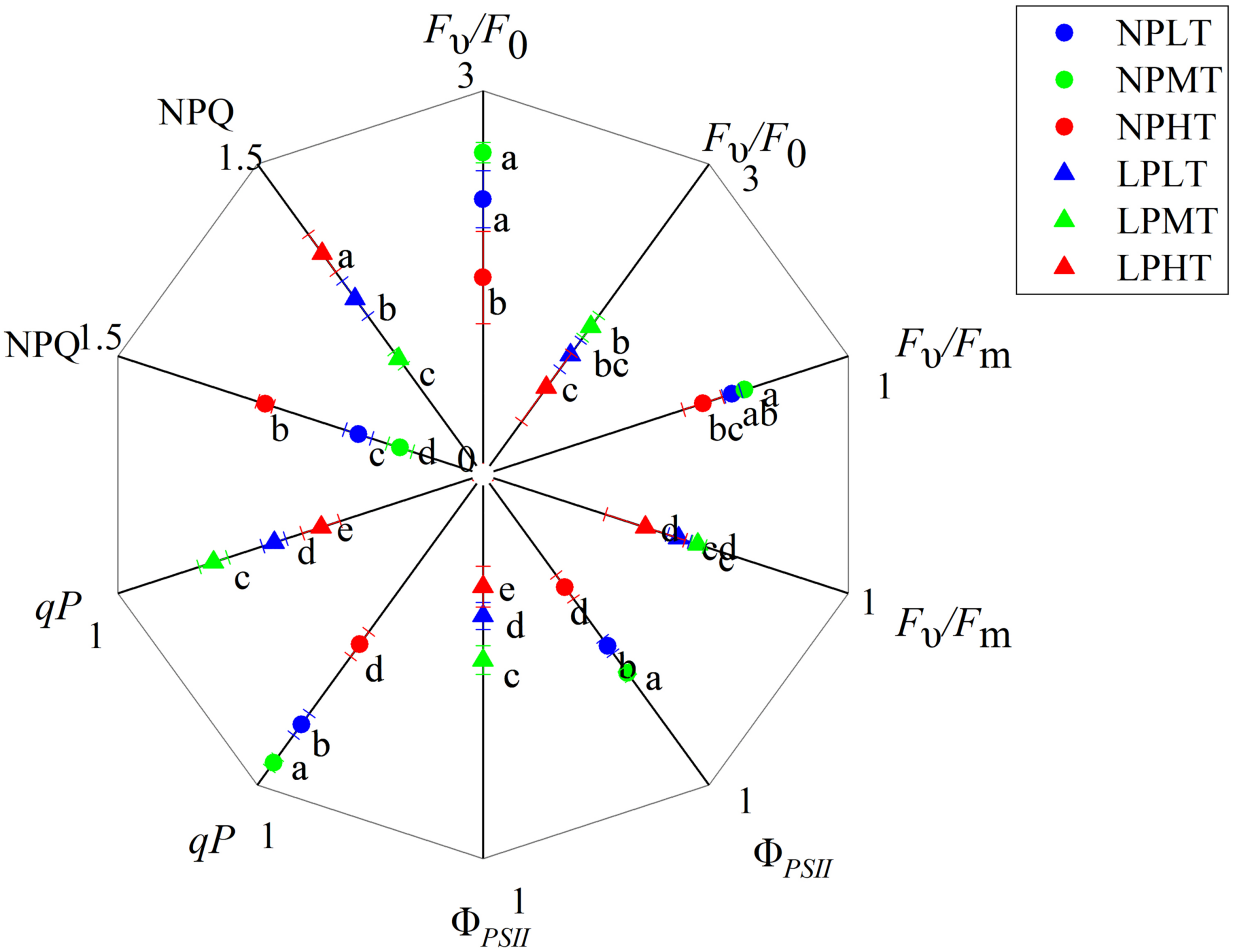
Effect of root zone temperature and phosphorus content on fluorescent characteristics. NT: 24°C, MT: 30°C, and HT: 36°C (root zone temperature); LP: 1.25μm and NP: 250μm (phosphorus content). Notations above bars of a, b, c, d, and e indicate homogeneous groups (*α*=0.05).

All statistical analyses were performed with the SPSS 20.0 (SPSS; Chicago, IL, United States). The Shapiro–Wilk was used to test the normality of data. Levene’s test was used to assess Homogeneity of Variances. Two-way ANOVA was used to examine the impacts of root zone temperature, phosphorus, and their interactions on dry mass accumulation, root morphology, root vitality, MDA content, phosphorus content, photosynthetic physiological parameters, and chlorophyll fluorescence (Table 1). One-way ANOVA was used to examine the impacts of the comprehensive effects of root zone temperature and phosphorus on dry mass accumulation, root morphology, root vitality, MDA content, phosphorus content, photosynthetic physiological parameters, and chlorophyll fluorescence (Table 2; Figures 1–5). Means are presented with standard error (SE). Differences were judged by the least significant differences test using a 0.05 level of significance. The figures were plotted using Origin2017 (OriginLab, Northampton, MA, United States).

**Table 1:** Two-way ANOVA of the effects of root zone temperature, phosphorus, and their interaction on maize growth.

	Phosphorus (P)		Root zone temperature (T)		T×P	
	Value of *f*	Value of *p*	Value of *f*	Value of *p*	Value of *f*	Value of *p*
Shoot dry mass	66.959	**<0.001**	47.004	**<0.001**	4.037	**0.046**
Root dry mass	7.720	**0.017**	50.665	**<0.001**	1.056	0.378
Root/shoot biomass	172.095	**<0.001**	8.024	**0.006**	0.351	0.711
Root surface area	23.175	**<0.001**	142.202	**<0.001**	0.679	0.526
Average root diameter	12.062	**<0.001**	50.559	**<0.001**	4.309	**0.039**
Total root length	20.722	**0.001**	51.293	**<0.001**	0.075	0.928
Root volume	6.196	**0.028**	79.775	**<0.001**	0.007	0.993
MDA content	150.257	**<0.001**	319.358	**<0.001**	6.455	**0.012**
Root vitality	116.405	**<0.001**	111.698	**<0.001**	5.354	**0.022**
Shoot phosphorus	301.013	**<0.001**	73.841	**<0.001**	44.829	**<0.001**
Root phosphorus	178.783	**<0.001**	51.184	**<0.001**	17.375	**<0.001**
Net photosynthetic rate	149.651	**<0.001**	58.159	**<0.001**	8.140	**0.006**
Transpiration rate	120.511	**<0.001**	198.165	**<0.001**	11.924	**0.001**
*F*_υ_/*F*_0_	70.881	**<0.001**	17.009	**<0.001**	1.134	0.354
*F*_υ_/*F*_m_	33.579	**<0.001**	9.333	**0.004**	0.121	0.887
Φ*_PSII_*	65.268	**<0.001**	64.500	**<0.001**	4.098	**0.044**
*qP*	99.621	**<0.001**	124.415	**<0.001**	4.708	**0.031**
NPQ	69.307	**<0.001**	110.005	**<0.001**	2.759	0.103

*Significant p values (p<0 0.05) are in bold*.

**Table 2:** Effect of root zone temperature and phosphorus content on the biomass accumulation and allocation in maize.

P supply	Root zone temperature	Shoot dry mass (*g*)	Root dry mass (*g*)	Root/shoot biomass (%)
NP	NT	0.789±0.038 b	0.168±0.008 bc	21.351±1.812 b
	MT	1.017±0.037 a	0.225±0.028 a	22.051±2.035 b
	HT	0.547±0.064 c	0.085±0.008 d	15.701±2.448 c
LP	NT	0.501±0.066cd	0.183±0.036 b	36.298±2.771 a
	MT	0.678±0.109 b	0.245±0.023 a	36.555±3.522 a
	HT	0.415±0.053 d	0.134±0.010 c	32.479±1.938 a

*NT: 24°C, MT: 30°C, and HT: 36°C (root zone temperature); LP: 1.25μm and NP: 250μm (phosphorus content). Notations of a, b, c, and d indicate homogeneous groups (α=0.05)*.

## Results

### Dry Mass Accumulation of Root and Shoot

Root zone temperature and phosphorus content significantly influenced dry mass accumulation of maize (Tables 1, 2). As the temperature of the root zone increased, the shoot dry mass and root dry mass increased first and then decreased. They were significantly increased under 30°C water temperature (MT) treatment and significantly decreased under 36°C water temperature (HT) treatment. Compared with the combination of normal phosphorus and root zone normal temperature treatment (NPNT), the dry mass of shoots and roots under normal phosphorus root zone medium temperature treatment (NPMT) increased by 28.90 and 33.93% (*p*<0.05), respectively, and under normal phosphorus root zone high temperature treatment (NPHT), they decreased by 30.67 and 49.40% (*p*<0.05), respectively. Compared with normal phosphorus treatment (NP), the shoot dry mass with low-phosphorus treatment (LP) was markedly reduced, whereas the root dry mass with LP treatment was increased. And the shoot dry mass was significantly influenced by the interaction between root zone temperature and phosphorus content (Table 1). The shoot dry mass had the highest level under the comprehensive treatment of NP and MT, and the lowest level under the comprehensive treatment of LP and HT. Compared with NPMT treatment, the shoot dry mass of the low-phosphorus root zone high temperature treatment (LPHT) was decreased by 59.19% (*p*<0.05). LP treatment significantly increased the root/shoot biomass, while under HT treatment the root/shoot biomass was reduced. Comparatively, the shoot dry mass and root dry mass were less affected by the temperature of the root zone under LP conditions.

### Root Morphological Traits

Root zone temperature and phosphorus content significantly affected root morphology, with a more pronounced effect of root zone temperature on root morphology (Figure 1; Table 1). LP and MT treatments significantly increased the root surface area and total root length. The average root diameter with MT treatment was significantly increased, but that with LP condition was decreased, and the combined effect of LP and HT treatments caused the lowest average root diameter. Regardless of P content, HT treatment significantly reduced root surface area, total root length, root volume, and average root diameter. Compared with NPNT treatment, the root surface area, total root length, root volume, and average root diameter of NPHT treatment were reduced by 28.30, 17.22, 24.72, and 6.33% (*p*<0.05), respectively.

### Root MDA Content and Root Vitality

LP and HT treatments significantly increased root MDA content, with a more marked effect of HT treatments on it. Under NP conditions, the MDA content of HT treatment was 149.07% higher than that of NT treatment (*p*<0.05). There was no significant difference between the root MDA content of MT treatment and NT treatment, regardless of P supply (Figure 2). LP and HT treatments significantly reduced root vitality. At normal P supply, root vitality was significantly increased by MT treatment. The root MDA content and root vitality were significantly influenced by the interaction between root zone temperature and phosphorus content (Table 1). A combination of LP and HT treatments caused the highest root MDA content and the lowest root vitality.

### Root and Shoot Phosphorus Content

Root zone temperature, phosphorus, and their interaction significantly affected the root and shoot phosphorus content, with a more significant effect of LP treatment on it (Figure 3, Table 1). As the temperature of the root zone increased, the root and shoot phosphorus increased at first but then decreased. The root and shoot phosphorus content had the highest level under the comprehensive treatment of NP and MT, and the lowest level under the comprehensive treatment of LP and HT. Compared with NPMT treatment, the root and shoot phosphorus content under LPHT treatment were reduced by 90.94 and 96.19% (*p*<0.05), respectively. Comparatively, the effects of HT and LP treatments on shoot phosphorus content were greater than that in roots, and the root and shoot phosphorus were less affected by the temperature of the root zone under LP condition.

### Net Photosynthetic Rate (P_n_) and Transpiration Rate (T_r_)

Root zone temperature, phosphorus, and their interaction significantly affected the net photosynthetic rate (P_n_) and the transpiration rate (T_r_; Figure 4; Table 1). As the temperature of the root zone increased, the P_n_ and T_r_ increased at first but then decreased. LP treatment significantly decreased P_n_ and T_r_, while MT treatment significantly increased them. A combination of LP and HT treatments caused the lowest P_n_ and T_r_. Compared with NPMT treatment, the P_n_ and T_r_ under LPHT treatment were reduced by 61.08 and 68.37% (*p*<0.05), respectively. Comparatively, LP treatment had a more pronounced impact on P_n_, and HT treatment had a more obvious impact on T_r_.

### Fluorescent Characteristics

LP treatment significantly decreased potential activity of PSII (*F*_υ_/*F*_0_) and maximum quantum yield of PSII photochemistry (*F*_υ_/*F*_m_). Compared with NPNT treatment, the (*F*_υ_/*F*_0_) and (*F*_υ_/*F*_m_) under the low-phosphorus root zone normal temperature treatment (LPNT) were reduced by 46.24 and 21.55% (*p*<0.05), respectively. While root zone temperature treatment had little effect on *F*_υ_/*F*_0_ and *F*_υ_/*F*_m_ (Figure 5). Comparatively, LP treatment had a more pronounced impact on *F*_υ_/*F*_0_ and *F*_υ_/*F*_m_. Root zone temperature and phosphorus content also have a significant effect on actual photochemistry efficiency (Φ*_PSII_*), photochemical quenching coefficient (*qP*), and NPQ, with a more pronounced effect of root zone temperature treatment on them. As the temperature of the root zone increased, the Φ*_PSII_* and *qP* increased at first but then decreased, while the NPQ decreased at first but then increased. LP treatment significantly decreased Φ*_PSII_* and *qP* and increased NPQ. A combination of LP and HT caused the lowest level of *F*_υ_/*F*_0_, *F*_υ_/*F*_m_, Φ*_PSII_*, and *qP*, and the highest level of NPQ.

## Discussion

Soil temperature and phosphorus are important environmental factors affecting the growth and development of maize. Extreme soil temperature or phosphorus stress has a significant impact on the dry mass accumulation and distribution in different growth stages of maize, especially the seedling stage (Tang et al., 2019; Xia et al., 2021b). At the same time, crops can cope with environmental stress by coordinating the growth of the root system and the above ground biomass (Tang et al., 2019; Xia et al., 2021b). In this study, low-phosphorus treatments and root zone warming treatments significantly affected the dry mass of maize seedling roots and shoots. Under low-phosphorus stress, the dry mass of the shoots was significantly decreased, while the root dry mass was increased. And the root/shoot biomass significantly increased under low-phosphorus (LP) treatment (Table 2). When the temperature of the root zone was increased appropriately, the dry mass of the roots and the shoots of the corn seedlings were significantly increased, while too high root zone temperature significantly reduced the dry mass, and the root dry mass declines more than the shoot part (Table 2). This indicated that phosphorus stress had a greater impact on the shoots, while root zone temperature had a greater impact on the root system. Root architecture significantly affects crop water and nutrient absorption. At the same time, crop root architecture has strong plasticity, and changes in soil environment and water and fertilizer resources will all have a significant impact on it (Tang et al., 2019; Xia et al., 2021b). Studies have shown that the dry mass accumulation and root volume of corn root system were significantly reduced under excessively high soil temperature (Xia et al., 2021b). Tang et al. (2019) found that phosphorus stress and salt stress significantly affect the dry mass and root architecture of maize seedlings. In this experiment, root zone temperature and phosphorus had important regulatory effects on the root architecture of maize seedlings. 30°C water temperature (MT) treatment caused a significant increase of root surface area, average root diameter, total root length, and root volume, but root development was significantly restricted under 36°C water temperature (HT) treatment. And the root surface area and root length significantly increased under LP conditions (Figure 1). Low-phosphorus treatment and root zone high temperature stress have different effects on roots. Crops ensure root nutrient absorption through higher root dry matter allocation and larger root absorption area under low-phosphorus conditions (Ding et al., 2021). At the same time, high temperature in root zone directly affects the root system and the root system is more sensitive to temperature, so it has a greater impact on the root dry mass and root architecture (Xia et al., 2021b). Phosphorus content and root zone temperature had a strong superimposing effect. Research on the occurrence and change mechanism of plant roots under low-phosphorus conditions were mainly focusing on the role of plant hormones in root formation, especially lateral roots and root hairs. Ethylene can increase plant root hair density and length under phosphorus stress (Lynch and Brown, 1997). Some experimental evidences indicate that auxin plays a key role in the process of controlling phosphorus starvation to cause the changes in root structure (Nacry et al., 2005), and high temperature conditions in the root zone may inhibit the effects of such hormones (Xia et al., 2021b). Under the comprehensive influence of LP and HT, the growth of maize seedlings was the worst. Under normal phosphorus level, proper root zone temperature can significantly promote dry mass accumulation of maize seedlings.

It has been known that soil temperature can regulate the absorption of phosphorus by plants. The previous studies suggested that appropriate increase of soil temperature can significantly increase phosphorus absorption and utilization efficiency (Ylivainio and Peltovuori, 2012). The findings of this study are in agreement with this statement that under 30°C root zone medium temperature treatment, the root and shoot phosphorus content of maize seedlings increased, and compared with the LPMT treatment, the NPMT treatment has a greater increase in the root and shoot phosphorus content. However, under the treatment of high temperature in the root zone of 36°C, the root and shoot phosphorus content of maize seedlings decreased, and under LPHT treatment, the root and shoot phosphorus content were the lowest (Figure 3). Under high temperature conditions in the root zone, the growth and development of the corn root system are severely impaired, the development of root hairs is impaired, and the root surface area decreases (Xia et al., 2021b). Since root hairs play an important role in the acquisition of plant phosphorus, this may be an important factor in the reduction of corn phosphorus content under HT conditions. LP treatment significantly reduced the root and shoot phosphorus content of maize seedlings, but under LP conditions, there was no significant difference in the root and shoot phosphorus content under different root zone temperature treatments. This indicates that the effect of phosphorus supply on the phosphorus content of maize seedlings is significantly greater than that of the root zone temperature. When maize show phosphorus deficiency symptoms, the phosphorus supply should be increased first. In the case of sufficient phosphorus supply, appropriately increasing the root zone temperature is an important way to increase the phosphorus content of the plant. In the corn production process, this effect can be achieved through different mulching methods.

The production of reactive oxygen species is a basic process in higher plants, which is used to transmit signal information in response to changing environmental conditions. However, their overproduction inhibits the growth of plant roots (Raja et al., 2017). MDA is the final product of membrane lipid peroxidation, and its content can reflect the extent to which plants suffer from adversity damage (Wen et al., 2012). Previous studies found that the MDA content increased significantly under temperature stress and phosphorus stress (Rohman et al., 2019; Xia et al., 2021a). This experiment found that the MDA content significantly increased under low-phosphorus stress and root zone high temperature, and the root zone high temperature treatment is more obvious, indicating that the membrane lipid peroxidation is obvious. The root zone high temperature condition destroys the balance between the production of active oxygen and the antioxidant defense system. This may lead to protein degradation, inactivation of key enzyme activities, changes in gene expression, and various metabolic pathways, leading to overall cell damage (Raja et al., 2017). However, the MDA content was not significant different compared with NT under the condition of medium temperature in the root zone, and membrane lipid peroxidation of corn roots was not obvious (Figure 2). The root system is an active absorption organ and a synthesis organ. Root vitality can reflect the absorption, synthesis, oxidation, and reduction capabilities of the root system (Jiang et al., 2017). In this experiment, MT caused a significant increase of root vitality, but the root vitality significantly reduced under the LP and HT conditions (Figure 2). At the same time, there was a significant interaction between root zone temperature and phosphorus stress. Under the combined action of NP and MT, the root vitality is significantly increased, which is beneficial to the growth and development of maize seedlings. Under the LP treatment, the excessively high root zone temperature significantly increased the root MDA content, and the root vitality was significantly reduced, which seriously affected the absorption function of the root system. This may be the physiological reason that the dry mass and phosphorus content of maize seedlings significantly decreased under the superimposed action of low phosphorus and root zone high temperature stress.

Photosynthesis is the basis for the accumulation of energy and material in crops, and the phosphorus content and root zone temperature have a significant impact on photosynthesis (Zhang et al., 2018; Xia et al., 2021a). In this experiment, LP stress and root zone warming treatment significantly affected the net photosynthetic rate and transpiration rate of maize seedlings. Among them, LP treatment had a greater impact on net photosynthesis rate, while root zone warming treatment had a greater impact on transpiration rate. The net photosynthetic rate was significantly reduced under the combined effects of low phosphorus and high temperature in the root zone (Figure 4). This is related to that phosphorus directly participates in many aspects of photosynthesis, including light energy absorption, Calvin cycle, assimilation formation and transportation, and regulation of the activity of some photosynthetic key enzymes (Fredeen et al., 1990). Under LPHT treatment, the shoot phosphorus content of maize was significantly reduced, resulting in a decrease in photosynthetic phosphorylation and a decrease in ATP production. Moreover, low phosphorus would cause a decrease in the content and activity of RUBISCO, a key enzyme of the Calvin cycle, and hinder the regeneration of RuBP (Rao et al., 1990). The transpiration rate of MT treatment was significantly increased, while that of HT treatment was significantly decreased under different phosphorus content conditions (Figure 4). Temperature is the most important factors affecting plant transpiration (Yang et al., 2012). The effect of soil temperature on transpiration is different from air temperature. Soil temperature may affect plant transpiration rate by regulating the water absorption of roots (Xia et al., 2021a). In this study, HT treatment had a significant impact on root surface area, root length, and root vitality, which might be an important reason for the decrease in transpiration rate.

Different scholars have different conclusions on the influence of chlorophyll fluorescence parameters under phosphorus stress. Studies have shown that LP had no effect on the photochemical mechanism of soybeans and sugar beets (Abadia et al., 1987; Lima et al., 1999), but LP can cause photoinhibition in corn and sunflowers, and cause the PSII reaction center to inactivate and the PSII photochemical efficiency to decrease (Jacob and Lawlor, 1993). In this experiment, (*F*_υ_/*F*_0_) and (*F*_υ_/*F*_m_) significantly decreased under LP treatment, while root zone warming treatment had little effect on (*F*_υ_/*F*_0_) and (*F*_υ_/*F*_m_; Figure 5). It showed that the PSII reaction center of maize seedlings was damaged and the photoinhibition was stronger under LP treatment. Φ*_PSII_* is the photochemical efficiency when the PSII reaction center is partially closed, and it can reflect the ratio of the energy used by photosynthetic electron transfer to the light energy absorbed by the leaves (Kalaji et al., 2017). Both LP treatment and root zone warming treatment had a significant effect on Φ*_PSII_* (Figure 5). Φ*_PSII_* was significantly increased under MT treatment, while LP and HT conditions caused excessive reduction of PSII, an increase in the degree of PSII closure, and a decrease in the efficiency of light energy conversion and electron transfer. *qP* and NPQ are two forms of chloroplast energy dissipation. *qP* is the part of the light energy absorbed by the PSII antenna pigment for photochemical reaction, and NPQ is part of the light energy absorbed by the PSII antenna pigment, which is dissipated in the form of heat, and it can prevent excessive light energy from damaging the PSII activity center (Guidi et al., 2019). MT treatment significantly increased *qP* under different phosphorus concentrations (Figure 5), indicating that when the root zone temperature moderately increased, the light energy utilization efficiency of maize seedlings was significantly increased. Under the conditions of LP and HT, *qP* was significantly decreased, while NPQ was significantly increased (Figure 5), indicating that the maize seedlings absorb excess light energy under LP and HT treatment, triggering their own photoprotection mechanism, and consume the excess excitation energy by means of heat dissipation. Phosphorus supply and root zone warming showed significant interactions on the net photosynthetic rate, transpiration rate, Φ*_PSII_*, and *qP* of maize seedlings (Table 1). Under the condition of sufficient phosphorus, appropriately increasing the root zone temperature can greatly improve the photochemical efficiency, light energy utilization, photosynthetic rate, and transpiration rate of maize seedlings. However, under the combined effect of low phosphorus and high temperature in the root zone, the photosynthetic transpiration rate was significantly reduced, which may be an important reason for the decrease in dry mass accumulation.

## Conclusion

Phosphorus deficiency and root zone temperature warming significantly affect the root and shoot growth of maize seedlings. Phosphorus deficiency has a greater impact on the shoots, while root zone temperature increase has a more significant impact on the root system. Appropriate increase in root zone temperature is beneficial to the growth of maize seedlings, while low phosphorus and root zone high temperature stress significantly reduced dry mass accumulation, total phosphorus content, and photosynthetic rate. There is a strong interaction between phosphorus content and root zone warming. Under the comprehensive influence of normal phosphorus supply and medium temperature in the root zone, the growth of maize seedlings is the best, while under the comprehensive influence of low phosphorus and high temperature in the root zone, the growth is the worst. In contrast, under the condition of phosphorus deficiency, root zone temperature changes have little effect on maize seedlings. In the case of sufficient phosphate fertilizer supply, appropriately increasing the soil temperature in the root zone is beneficial to increase the absorption and utilization of phosphorus by plants and promote the growth and development of maize seedlings. In the management of corn production, when the supply of phosphate fertilizer is sufficient, various mulching methods, such as mulching and straw mulching, can be used to appropriately regulate the root zone temperature, give full play to the coupling effect of root zone temperature and phosphorus, and improve the absorption and utilization efficiency of phosphorus by plants.

## Data Availability Statement

The raw data supporting the conclusions of this article will be made available by the authors, without undue reservation.

## Author Contributions

ZX, GZ, SZ, QW, and YF: investigation. ZX: writing—original draft preparation. HL: writing—review and editing. All authors have read and agreed to the published version of the manuscript.

## Funding

This research was funded by the Natural Science Fund of China (grant number 31771724).

## Conflict of Interest

The authors declare that the research was conducted in the absence of any commercial or financial relationships that could be construed as a potential conflict of interest.

## Publisher’s Note

All claims expressed in this article are solely those of the authors and do not necessarily represent those of their affiliated organizations, or those of the publisher, the editors and the reviewers. Any product that may be evaluated in this article, or claim that may be made by its manufacturer, is not guaranteed or endorsed by the publisher.
